# Editorial: Insights in cellular neurophysiology: 2021

**DOI:** 10.3389/fncel.2022.1037824

**Published:** 2022-09-27

**Authors:** Enrico Cherubini, Arianna Maffei, Daniela Tropea

**Affiliations:** ^1^European Brain Research Institute, Rome, Italy; ^2^Department of Neurobiology and Behavior, Stony Brook University, Stony Brook, NY, United States; ^3^Institute of Neuroscience, Trinity College, Dublin, Ireland

**Keywords:** developing neocortex, electrical synapses, X-rays, hippocampus and striatum, spinal cord injury, dorsal raphe, circadian rhythms, olfaction

In the framework of Frontiers' initiative to highlight the latest advancements in Neuroscience, this Research Topic is focused on new insights, recent developments and major accomplishments achieved in the Cellular Neurophysiology field. The goal of this special edition is to shed light on the progress made in the past decade, and on future challenges, providing a thorough overview of the state of the art of this stimulating area of research.

This Research Topic gathers contributions (four reviews and twelve research articles) from editorial board members of the Cellular Neurophysiology Section of the Journal.

## Reviews

Luhmann summarizes novel technologies used to explore the developing cerebral cortex in newborn rodents. In the past decade, high-density multi-electrode arrays and genetically encoded calcium indicators have been used to detect the activity of large neuronal ensembles, to characterize their connectivity and architectural layering which changes in an add-on daily basis process. Moreover, opto-chemogenetic approaches allowed the identification of transient cortical circuits, hub neurons and their role in generating spontaneous and sensory evoked activities. At early stages of development, network synchronization is facilitated by the early depolarizing action of GABA and by electrical synapses.

In mature neurons, electrical synapses are not only responsible for network synchronization but, as highlighted by Vaughn and Haas, they provide spike-dependent inhibition, acting as low-pass filters that can preferentially transfer the slow spike after-hyperpolarization. Furthermore, gap junctions are instrumental in processing a variety of neuronal signals. They have been shown to drive asynchronous firing, regulate excitation *via* shunting inhibition, and improve the signal to noise ratio. Computational modeling and imaging approaches will certainly contribute to a better understanding of how gap junctions integrate neuronal activity in a network.

The prefrontal cortex plays a key role in neuronal circuits responsible for social status, a major social determinant of health in primates (Wilkinson et al., 1998). This leads to hierarchical behavior, which represent a universal feature among animal species from insect to fishes, including rodents and primates. It is crucial for animals' survival, and is regulated by the interaction of neuroendocrine factors and neuronal circuits. The current “prefrontal-centric” view of social hierarchy behavior has been expanded in Ferreira-Fernandez and Peça's review, in which, on the basis of connectivity data, possible interactions at macro, micro and mesoscale levels of the prefrontal cortex with other brain regions are discussed.

The biological effects of X-rays, a form of high energy electromagnetic radiation that can penetrate tissues more readily than light thus affecting neuronal and behavioral functions in animals, have been reviewed by Mantraratnam et al. who have examined sensory effects of X-rays mediated by radiolysis of water and generation of reactive oxygen species in a variety of animals. The authors focused mainly on immediate appetitive and consummatory behaviors, radiotaxis (a locomotor movement toward the source of X-rays including flight in insects), arousal and olfactory responses.

## Research articles

Interferon γ (IFN-γ) is a cytokine with neuromodulatory properties. In particular, IFN-γ enhances GABAergic transmission in the hippocampus (Flood et al., 2019). In view of the key role of GABA in shaping neuronal circuits early in development, Döhne et al. investigated whether IFN-γ affects miniature GABAergic currents in layer 5 pyramidal neurons of developing rat somatosensory cortex. IFN-γ enhanced the frequency and amplitude of spontaneous GABA_A_-mediated events. While the increase in frequency was dependent on nitric oxide (NO) and guanylate cyclase, the amplitude relied on protein kinase C. In addition, IFN-γ shifted paired-pulse ratio toward facilitation in a NO-independent manner.

Piepgras et al. examined the effects *Clostridium botulinum* C3 transferase, C3BOT (an exoenzyme known to inhibit Rho-dependent signaling cascades) and the 26mer peptide derived from full length protein in regulating glutamate transporter EAAT3 in primary murine hippocampal neurons. Both proteins provide neuroprotection by promoting upregulation of glutamate uptake, an effect that is prevented by tyrosine kinase inhibitors. In pathological conditions, the C3-mediated increase of glutamate clearance would limit glutamate spillover and excitotoxicity.

Kamiya used the NEURON simulator to revise the model proposed by Alle and Geiger (2006) on excitatory presynaptic potentials (EPreSPs) at mossy fibers-CA3 synapses in the hippocampus. The model described by the author incorporated active axonal sodium, potassium and calcium conductance to affect transmitter release at axon terminals. In addition to their canonical mode of signaling, somato-dendritic depolarization can affect action potential amplitude and information transfer in neuronal circuits through passive propagation. Furthermore, he showed that activation of axonal GABA_A_ receptors by EPreSPs generated by spillover of GABA from adjacent synapses reduced spike's amplitude *via* shunting inhibition.

Anandamide (AEA) and 2-arachidonylglycerol (2-AG), endogenous ligands of endocannabionoid receptors, regulate synaptic transmission in several brain areas. Recent evidence suggests that fatty acid-binding protein 5 (FABP5) controls synaptic 2-AG signaling at excitatory synapses in the dorsal raphe (Haj-Dahmane et al., 2018). Fauzan et al. investigated whether a similar effect occurs in the striatum, which expresses high levels of FABP5 mainly in astrocytes. They found that FABP5 deletion impairs tonic 2-AG and AEA signaling at medium spiny neurons synapses and alters short-term synaptic plasticity in the striatum.

Dysregulation of the activity of serotoninergic (5-HT) neurons in the dorsal raphe nucleus (DRN) is responsible for emotional disorders. The activity of 5-HT neurons is regulated by α1-adrenoreceptors activated by the noradrenergic input from locus coeruleus. In brain slice preparation, Wang et al. demonstrated that inhibition of K^+^ currents from three K^+^ channel families, A-type, Kv7/KCNQ and SK channels, contributes to spontaneous firing triggered in DRN 5-HT neurons by activation of α1-adrenoreceptors agonist phenylephrine.

Neurons in the rostral nucleus of the solitary tract (rNST), which receive taste information from the tongue, project to brainstem relay nuclei along the taste pathway. Park et al. used anterograde horseradish peroxidase labeling and post-embedding immunogold staining for glutamate to quantify, at the electron microscopic level, rNST terminals in parabrachial and medullary reticular formation nuclei, in order to examine how sensory information from rNST is processed.

Spinal cord injury (SCI) is one of the major causes of disability whose treatment is very limited. Among different neuronal populations, interneurons play a key role in circuit reorganization and partial recovery in less severe, anatomically incomplete SCI. Vargova et al. developed a new method, in rodents, aimed at enhancing the intrinsic regenerative properties of spinal cord interneurons, with the potential of expanding its use for SCI treatment.

In the spinal cord, substantia gelatinosa neurons of the dorsal horn are referred as “central gate” for transmission and regulation of nociceptive information. Zhu et al. demonstrated that a subset of these neurons, with particular morphological and electrophysiological characteristics, exhibit rebound depolarization (RD). RD is a transient membrane depolarization following hyperpolarizing pulses that is regulated by cyclic nucleotide and T-type calcium channels, and can transform inhibitory signals into excitatory ones. RD-expressing neurons receiving monosynaptic as well as polysynaptic inputs from Aδ and C fibers, differentially process somatosensory information along the pain pathway.

CLC-3-associated Cl^−^/H^+^ exchangers are expressed in multiple endosomal compartments, and regulate pH and [Cl^−^] *via* stoichiometrically coupled exchange of two Cl^−^ and one H^+^. Downregulation of CLC-3 alters pain perception in mice (Pang et al., 2016), suggesting a role of Cl^−^/H^+^ exchangers in pain regulation. Sierra-Marquez et al. analyzed the involvement of CLC-3 Cl-/H^+^ transport in nociceptive pathways of the spinal cord. Genetic ablation of CLC-3-associated Cl^−^/H^+^ exchanger did not modify the excitability of DRG neurons, but enhanced microglia activation within spinal tissue, indicating that CLC-3 Cl^−^/H^+^ transport is needed for maintaining neuroglia homeostasis.

Brain stimulation devices are increasingly used to treat various forms of brain disorders. New insights into the mechanisms of bioelectronics in medicine have been provided by Brambilla-Pisoni et al. who used a non-invasive auricular transcutaneous vagus nerve stimulation (atVNS) to study its effects on cognitive processes in *naïve* CD-1 mice. Delivery of atVNS immediately, but not 3 h after the familiarization phase, induced a clear reorganization of the network leading to the enhancement of memory consolidation in a novel object recognition test and a re-distribution of the immediate early gene c-FOS.

In mammals, the circadian clock synchronizes physiological functions to day-night rhythms (Neumann et al., 2019). Disruption of circadian rhythms leads to chronic diseases including cardio-metabolic and neurodegenerative disorders, mainly *via* oxidative stress. Srimani et al., used three different cell lines and two circadian luminescence reporter systems to show that two lichen secondary metabolites, evernic and usnic acids (EA and UA), have marked neuroprotective and antioxidant effects. Both EA and UA significantly lower amplitudes and accelerate the dampening of cellular circadian rhythms.

In the last paper of this Research Topic, Miazzi et al. used functional imaging and analysis of calcium dynamics to detect how odors are processed in odorant receptors localized on olfactory sensory neurons (OSNs) in *Drosophila melanogaster*. Insects need chemoreception to identify food, mates, oviposition sites and, for their survival, to avoid exposure to harmful perils. In the fly, OSNs are expressed on two easily accessible organs: the antenna and the maxillary palps. To study odor-induced responses in OSNs under natural conditions, the authors used an *in vivo* and an *in vitro* preparation of isolated vital *Drosophila* OSNs from the antenna.

We hope that papers included in this Special Topic can stimulate further studies leading to new advances in the fast-growing field of Cellular Neuroscience.

## Author contributions

All authors contributed to the article and approved the submitted version.

## Funding

This research was funded by Fondo Ordinario Enti (FOE D.M 865/2019) to EC.

## Conflict of interest

The authors declare that the research was conducted in the absence of any commercial or financial relationships that could be construed as a potential conflict of interest.

## Publisher's note

All claims expressed in this article are solely those of the authors and do not necessarily represent those of their affiliated organizations, or those of the publisher, the editors and the reviewers. Any product that may be evaluated in this article, or claim that may be made by its manufacturer, is not guaranteed or endorsed by the publisher.

## Abbreviations

AEA: anandamide
2-AG: 2-arachidonylglycerol
atVNS: auricular transcutaneous vagus nerve stimulation
C3BOT: *Clostrdium botulinum* C3 transferase
DRN: dorsal raphe nucleus
EA: evernic acid
EAAT3: excitatory amino acid transporter 3
EPreSPs: excitatory presynaptic potentials
FABP5: fatty acid-binding protein 5
IFN-γ: Interferon γ
NO: nitric oxide
OSNs: olfactory sensory neurons
rNST: rostral nucleus of the solitary tract
SCI: Spinal cord injury
UA: usnic acid.
